# Supplementary material for: Tracking the COVID-19 pandemic in Australia using genomics
Source: Nat Commun. 2020 Sep 1;11:4376. doi: 10.1038/s41467-020-18314-x (PMC7462846; doi:10.1038/s41467-020-18314-x)
Supplement: Supplementary file 1 — Supplementary Information [file 41467_2020_18314_MOESM1_ESM.pdf]

Supplementary Information

Seemann et al. **Tracking the COVID-19 pandemic in Australia using genomics**

Supplementary Methods

Setting, data sources and COVID-19 genomics response group

In Australia, all cases of COVID-19 are immediately notified to public health authorities in each State or Territory. The Victorian Infectious Diseases Reference Laboratory (VIDRL) is the public health virology reference laboratory for the State of Victoria in Australia, covering a resident population of approximately 6.24 million. All primary samples testing positive for SARS-CoV-2 by RT-PCR at diagnostic laboratories are forwarded to VIDRL for additional confirmatory RT-PCR testing, as previously described.[1] Concurrently, primary samples or extracted nucleic acid are sent to a second reference laboratory in Victoria, the Microbiological Diagnostic Unit Public Health Laboratory (MDU PHL) for WGS and bioinformatic analysis. We conducted a retrospective, observational study of all patients in Victoria with confirmed COVID-19 with an onset of infection prior to 14 April 2020. Detailed demographic and risk factor information on each case was obtained from the Victorian Department of Health and Human Services (DHHS) and collected through individual case interviews using standardized case report forms. Data obtained included gender, age, date of symptom onset, and risk factors for infection including whether the case was a healthcare worker, date and location(s) of recent travel, and contact with any other suspected or confirmed cases of COVID-19 prior to illness onset. Epidemiological clusters were defined as those clusters containing three or more cases with a common source exposure (e.g. healthcare facility; social venue; cruise ship). Geographic region of travel was categorized using the Standard Australian Classification of Countries, 2nd edition[2].

To rapidly implement SARS-CoV-2 genomic analysis into local public health responses, a COVID-19 genomics response team was convened. This included representatives from the state health department, virology laboratory, the public health genomics laboratory (genomic epidemiologist, bioinformaticians and medical microbiologists) and academics with expertise in statistical genomics. Laboratory and bioinformatic workflows were developed to enable large-scale rapid genomic processing of samples, enabling sequencing and bioinformatic analysis of 96 samples in an approximately 45-hour time period. The response team held online meetings (weekly plus *ad hoc* as required) to enable interactive reporting of genomic epidemiological analyses and facilitate rapid translation of genomic findings into public health responses.

Genomic sequencing and bioinformatic analysis

RNA was extracted from 200ul of viral transport media from samples testing positive for SARS-CoV-2 on the QIAsymphony using the DSP Virus/Pathogen Mini Kit (Qiagen) and eluted into 60µl elution buffer as supplied in the kit by the manufacturer. Complementary DNA (cDNA) was prepared from RNA extracts as follows: 11µl template RNA, 1ul 50µM random hexamers (Thermo Fisher Scientific, Waltham, MA, USA), 1µl 10 mM dNTPs (Thermo Fisher Scientific) were mixed together and incubated at 65°C for 5 sec. To the annealed template RNA, 4µl SuperScript IV buffer (Thermo Fisher Scientific), 1µl 100mM DTT, 1µl RNaseOUT (Thermo Fisher Scientific) and 1µl SuperScript IV (Thermo Fisher Scientific) was added and the reaction incubated at 42°C for 50 minutes followed by 70°C for 10 minutes. Tiled amplicon PCR of cDNA was performed using two ARTIC primer pools (version 1 or version 3, https://github.com/artic-network/artic-ncov2019/tree/master/primer_schemes/nCoV-2019) employing published protocols (https://www.protocols.io/view/ncov-2019-sequencing-protocol-bbmuik6w) using the following reaction mix: 2.5µl template cDNA, 5µl 5x Q5 reaction buffer (New England Biolabs, Ipswich, MA, USA), 0.5µl 10mM dNTPs (Thermo Fisher Scientific), 0.25µl Q5 Hot Start DNA polymerase (New England Biolabs), 3.6µl primer pool 1 or 2 (10µM). 10µM primer pools were prepared from 'lab ready' 100µM stock solutions supplied by Integrated DNA Technologies (Coralville, Iowa, USA); refer to Supplementary Dataset 3. Cycling conditions were 98°C for 30 seconds, followed by 30 cycles of 98°C for 15 seconds, 65°C for 5 seconds. 'Pool 1' and 'Pool 2' reactions were combined and cleaned up using a 1:1 ratio of SPRI beads (Beckman Coulter, Brea, CA, USA) followed by elution in 30ul EB buffer (Qiagen). Amplicons were quantified using the Quant-iT High Sensitivity DNA assay (Thermo Fisher Scientific). Sequencing libraries were prepared using 1ng tiled Amplicons with the Nextera XT DNA library Prep Kit (Illumina, San Diego, CA, USA) and sequenced on either the NextSeq500/550 or iSeq100 (Illumina) using 150bp paired-end reads according to manufacturer's protocols.

Consensus sequence generation

Paired Illumina reads were aligned to the 29903nt Wuhan reference (Genbank MN908947.3) using *minimap2* (v2.17, options "-ax sr")[3]. The output of *samtools*[4] *mpileup* (v1.10, options "-aa -d 0 -A -B -Q 0") was then used by *ivar consensus*[5] (v1.2.1, options "-m 10 -t 0.9 -n N"). We applied quality control checks on generated sequences, requiring ≥80% genome recovery, ≤25 single nucleotide polymorphisms (SNPs) from the Wuhan reference, and ≤300 ambiguous bases (~1% of the genome) for sequences to 'pass' QC.

Phylogeographic context

To examine the distribution of the cases in this study in an international context we constructed a phylogenomic tree from our 903 genomes and 991 publicly available genomes from GISAID [https://www.gisaid.org/] as of 1st May 2020. The 991 GISAID genomes were randomly chosen from 14,451 non-Australian genomes having minimum length of 29,000-nt and less than 5% unknown basepairs. An approximate distance matrix was generated for Figure 2 (main manuscript) using MASH[6] (v2.2.2, options "triangle -s 5000 -k 15") and neighbor-joining tree computed using Quicktree[7] (v2.5, options "-in m -out t").

Phylogenomic analysis

For phylogenetic analysis, a single sequence was selected to represent each patient based on the best sequencing QC parameters. The Wuhan-1 reference genome was included as an outgroup to add directionality to the tree because it represents the oldest sequenced case. A multiple sequence alignment was generated using MAFFT[8] (v7.453, options "--auto"). Alignment cleanup was performed using *arbow* (v0.4.0, options "-x 0.998 -mm 20"; https://github.com/MDU-PHL/arbow). Briefly, this trims the 5' and 3' UTR regions, removes sites with too many gaps, removes sites with one singleton minor allele, and generates a maximum likelihood tree using IQ-Tree[9] (v1.6.12, options "-mset HKY,TIM2,GTR -mfreq F -mrate G,R -alrt 1000 -bb 1000")[7.] Results were visualized using FigTree[10] and a locally-developed tool for interactive visualization of combined phylogenetic and epidemiologic data via a secure web portal.

Cluster discovery

Genomic clusters were determined using ClusterPicker[11] (v1.2.3, options "70.0 95.0 0.0004 15"); settings were initially selected and optimized based on a small number of well-defined epidemiological clusters, then applied to the whole dataset. Concordance between genomic and epidemiologic clusters was assessed by identifying the intersection between genomic and epidemiologic clusters, and discrepancies were subsequently discussed with the Victorian Department of Health and Human Services to further clarify the available epidemiologic data. Lineages were assigned according to the recent proposed nomenclature[12] using *pangolin* [https://github.com/hCoV-2019/pangolin].

Quantifying the contribution of importation events to SARS-CoV-2 in Victoria

We analyzed the complete alignment in BEAST2.5[13] using a GTR+Γ substitution model a Γ distributed prior on the evolutionary rate with mean 1×10^{-3} subs/site/year and variance of 10%, as estimated recently[14]. To approximate the posterior distribution, we set a Markov chain Monte Carlo (MCMC) of 2×10^{8} steps, sampling every 1×10^{5} steps. We extracted 1,000 trees from the posterior and inferred a number of statistics using NELSI[15]. Considering the posterior distribution of trees ensures that we incorporate phylogenetic uncertainty into these analyses.

We detected local transmission lineages as monophyletic groups of at least two samples from individuals with no known travel history and presumed to have been locally infected[16]. Cases with no known recent travel history that fall outside of transmission lineages are referred to as singletons (Figure S4). These statistics are informative about importation events and account for phylogenetic uncertainty, but do not represent a formal migration model. We selected this approach because our intensive sampling of Victorian data would lead to sampling bias errors in formal phylogeographic methods[17].

Intra-host diversity

Intra-patient sequence variability was assessed by comparing consensus sequences from different samples from the same patient (where sequences had passed QC), as measured by single nucleotide polymorphism (SNP) distances. Only pairwise aligned sites containing A, C, G, or T were counted. The distribution of SNP distances within-patient was compared to SNP distances between a randomly chosen subset of genomes from different patients in the main dataset. We elected to compare consensus sequences, rather than raw reads, as there is currently uncertainty about frequency and signatures of RNA degradation or other sequencing artefacts, which could potentially introduce minor allelic variants that do not reflect true differences between repeat sequences from the same patient *in vivo*[18].

Estimation of population parameters

We estimated a phylogenetic tree using maximum likelihood, as implemented in IQ-Tree[9] under the GTR+Γ substitution model. To assess temporal signal, we conducted a root-to-tip regression using TempEst[19]. The slope for the root-to-tip regression was 1.05×10^{-3} subs/site/year, the X-intercept 2019.92. Because these values are similar to previous estimates[20], we considered them to indicate temporal signal in the data. Phylodynamic analyses are sometimes sensitive to underlying population structure, which we assessed by repeating our analyses on two subsets, one that involved travel-associated samples only, and one where these samples were excluded. These subsets revealed nearly identical estimates to those from the complete data, indicating that there is not sufficient differentiation between these groups so as to warrant separate analyses. In general, phylodynamic analyses assume a well-mixed population, which is not the case in our analyses due the large number of importations and heterogeneity among overseas outbreaks. Our population dynamic inferences correspond to averages over the diversity sampled in Victoria, and should not be interpreted to represent transmission dynamics within outbreak clusters. Our phylodynamic models and configurations were:

Constant coalescent exponential and birth-death

These models posit that epidemiological dynamics are governed by a constant R_e value over time. The coalescent exponential has two compound parameters; the growth rate with a Laplace prior with μ=0.0 and scale=100, and the scaled population size with an exponential prior with mean=1000. The birth-death model has a different parameterisation[21]; R_e with a lognormal prior with μ=0.8 and σ=0.5 and sampling probability with a β distribution with α=1.0 and β=1.0. In both of these models, the epidemic doubling time can be estimated as log(2) / growth rate. The sampling probability represents the probability that a case will be successfully sequenced. We allowed the sampling proportion to vary before and after the first Victorian sample to represent local sequencing effort.

Birth-death skyline

The birth-death skyline model[21], relaxes the assumption of a constant R_e over time, but it requires careful consideration to its configuration. We specified two time-intervals, during which R_e was constant. Although the model allows the inclusion of more time-intervals, our aim was to assess the single time point with strongest evidence for a change in R_e, as a means to determining whether travel and social distancing restrictions had an effect on this parameter. The interval break-point time was estimated from the data, such that it corresponds to the point in time with strongest evidence for a change in R_e. For the interval breakpoint we set a uniform prior between the most recently collected genome, 13 April, and 31 January. Our priors for R_e and the sampling proportion were the same as those for the birth-death model.

For all our Bayesian phylodynamic analyses we used an uncorrelated lognormal molecular clock model, with an informative Γ prior on the mean rate with mean 1×10^{-3} subs/site/year and variance of 10%, as estimated recently[14]. Importantly, phylodynamic methods require a prior assumption about at least one individual parameter. To this end, we fixed the duration of infection to 9.68 days, as estimated via epidemiological modelling in a recent DHHS report[22].

Supplementary Tables

Supplementary Table 1. Demographic and risk factors data for Victorian COVID-19 cases and those with available sequence data, 19 January to 14 April 2020

	All cases (n=1333)	Cases with available sequence data[a] (n=903)	*p*-value
**Case demographics**			
Age (median, IQR)	47 years (29-61)	46 years (29-60)	0.119
Sex (n, % male)	631/1205 (52.4%)	473/883 (53.6%)	0.595
Location of residence (n, % metro)	902/1185 (76.1%)	664/873 (76.1%)	1.000
Healthcare worker (n, %)	163/1333 (12.2%)	109/903 (11.9%)	0.947
**Putative source of acquisition (n, %)**			
Overseas travel	827 (62.0%)	557 (61.7%)	
Contact with known case	360 (27.0%)	260 (28.8%)	0.528
Unknown source	134 (10.1%)	81 (9.0%)	

[a] Cases with at least one sequence which met quality control metrics
Denominators reflect number of cases where data were available for that characteristic
Metro, metropolitan; IQR, inter-quartile range
Variables compared with chi square (sex, location of residence, healthcare work and putative source of acquisition) or Wilcoxon-rank sum (age).

Supplementary Table 2. Diversity of SARS-CoV-2 sequences within and between hosts

Characteristic	Result
No. of patients / sequences	63 patients / 145 sequences
No. of sequences per patient (median, range)	2 (2-5)
SNP differences within a pair of sequences from same patient (median, range)	0 (IQR 0-0, range 0-18)
SNP differences between patients (median, IQR)	11 SNPs (IQR 7-15)

IQR, inter-quartile range

Supplementary Figures

Supplementary Figure 1: Numbers of patients and sequences included in each analysis

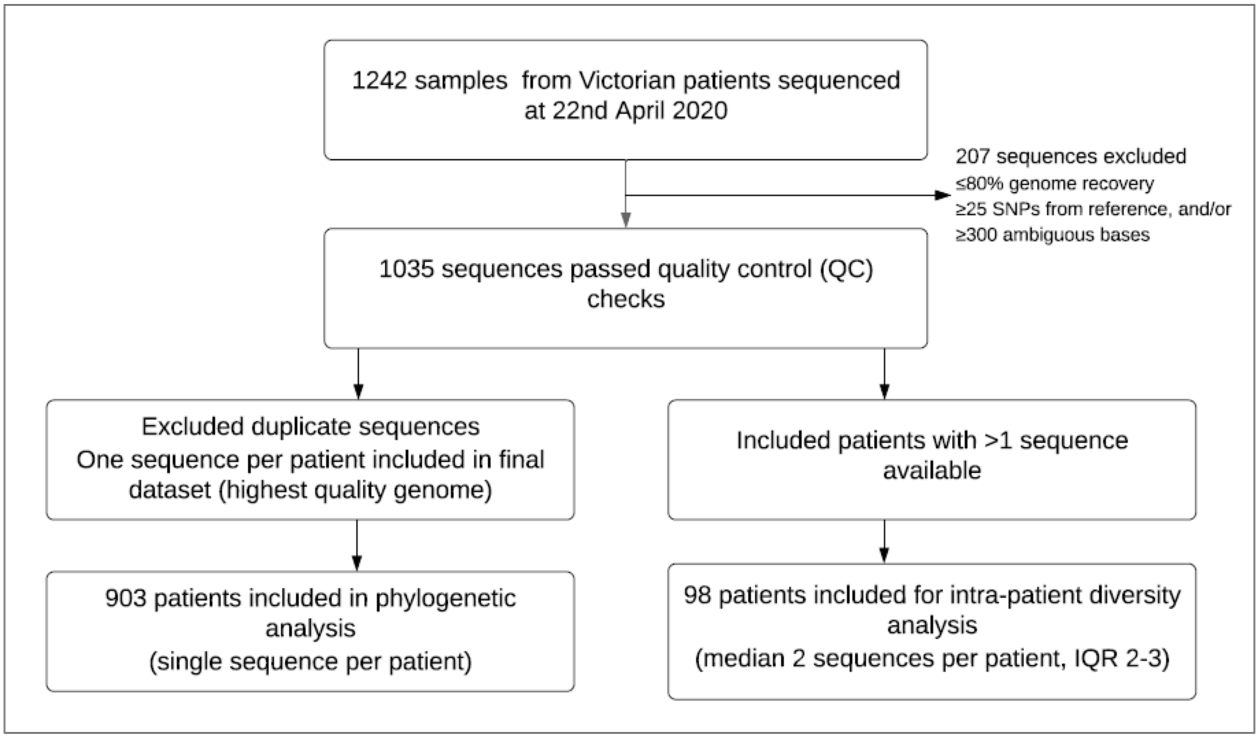

QC, quality control; IQR, inter-quartile range; SNP, single nucleotide polymorphism.

Supplementary Figure 2: PCR cycle threshold (Ct) value by sequence quality control outcome (QC PASS/FAIL)

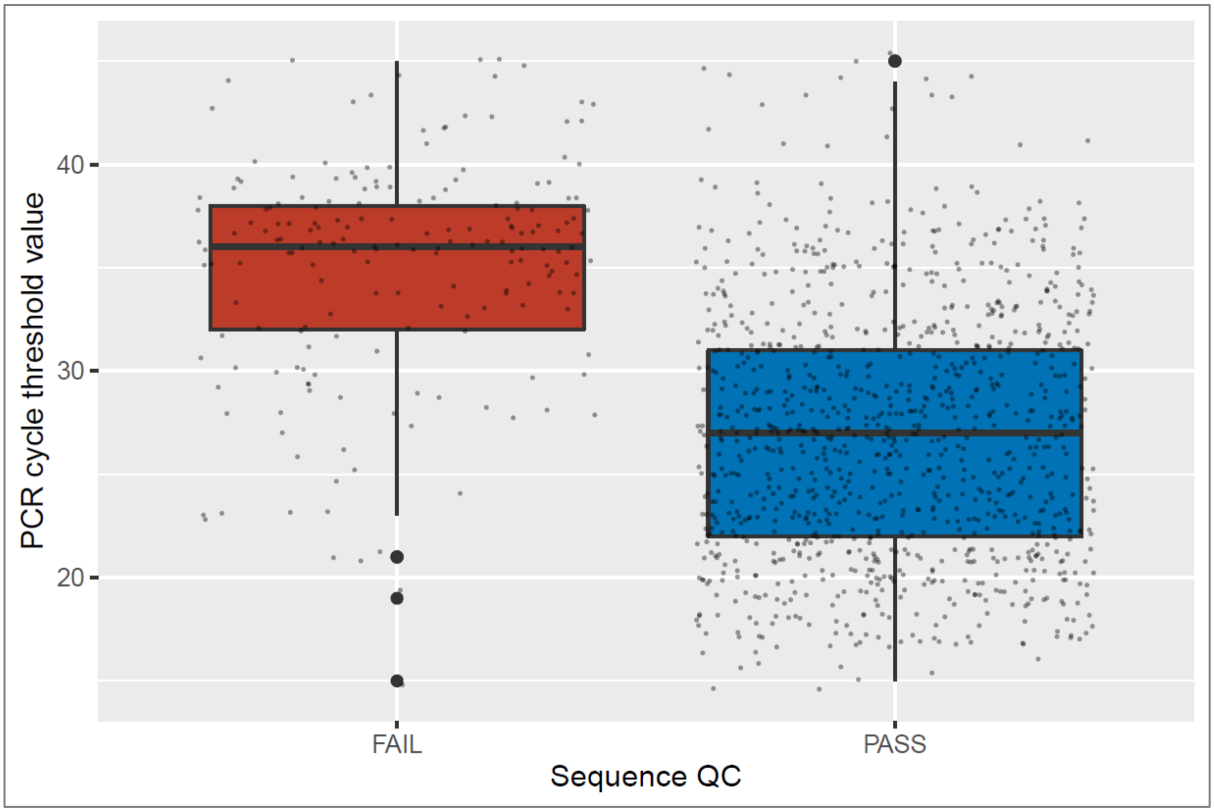

For each box in plot, middle line represents median; upper box margin represents third quartile, lower box margin represents first quartile, distance between upper and lower lines represent interquartile range (IQR), whiskers represent 1.5x IQR. Each small dot represents PCR Ct value for a single sequence (horizontal spread for ease of visualization only), single replicate only. Large dots represent outliers beyond 1.5x IQR. Sequence QC FAIL: n=218 (minimum Ct 15, maximum Ct 45); PASS: n=1085 (minimum Ct 15, maximum Ct 45).

Supplementary Figure 3. Genomic diversity within and between patients

Supplementary Figure 3a. Pairwise SNP distance distribution across included samples

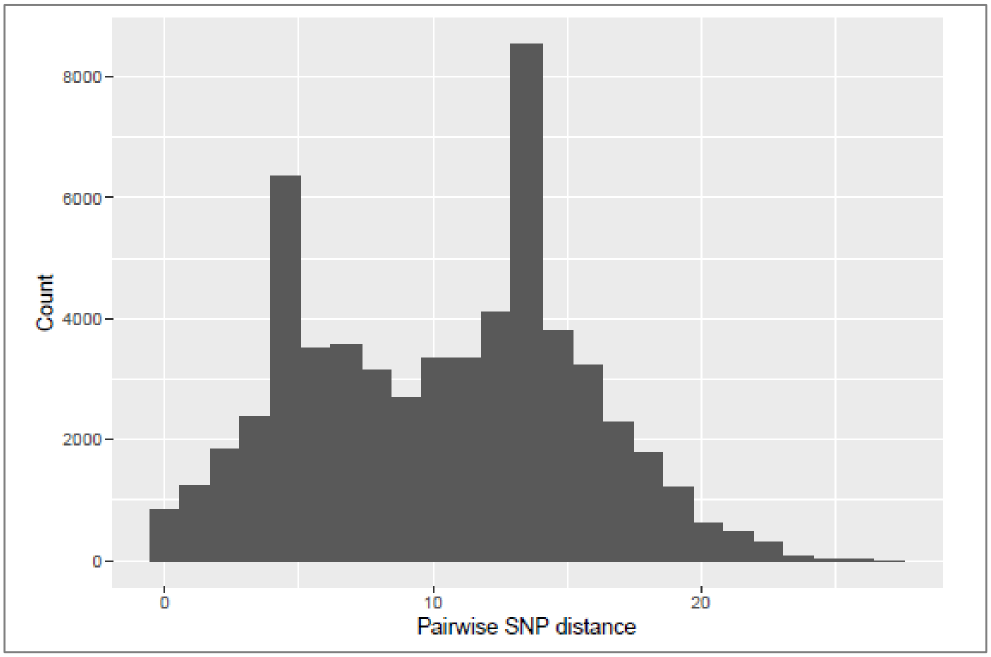

For this analysis, a random subset of all patient samples included in the dataset was selected (n=200). The genomic relatedness of sequences included in the dataset is assessed by comparing each isolate to every other isolate in a pairwise fashion, and tallying the number of SNPs for each pair.
SNP, single nucleotide polymorphisms.

Supplementary Figure 3b. Median SNP distance of each sample to all other included samples

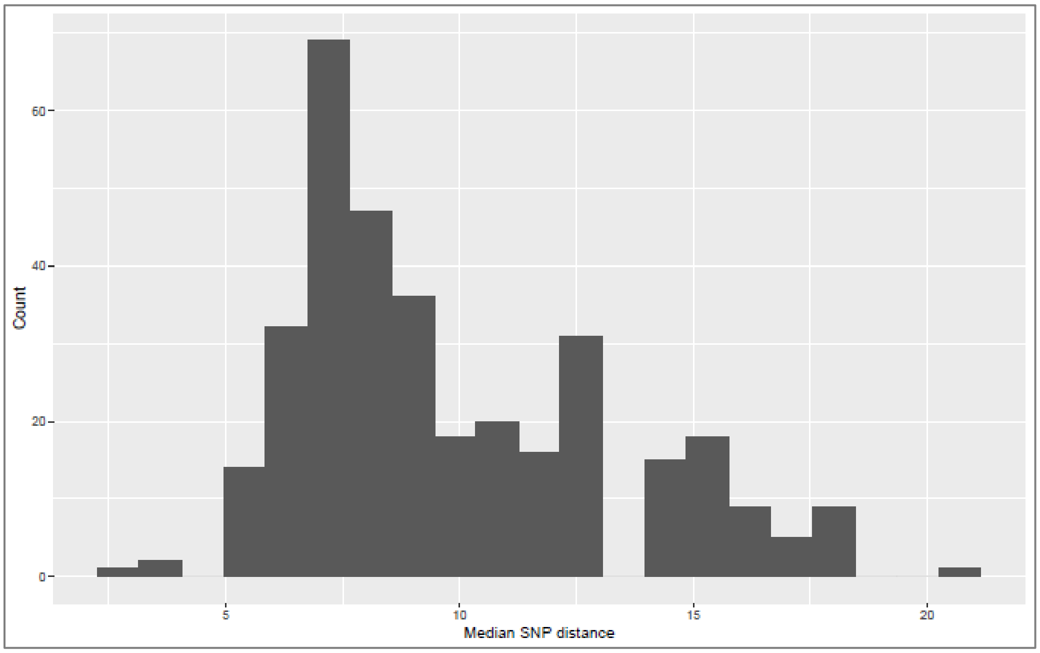

See Supplementary Figure 3a for description of included samples.

Supplementary Figure 3c. Observed pairwise SNP distances between samples from the same patient

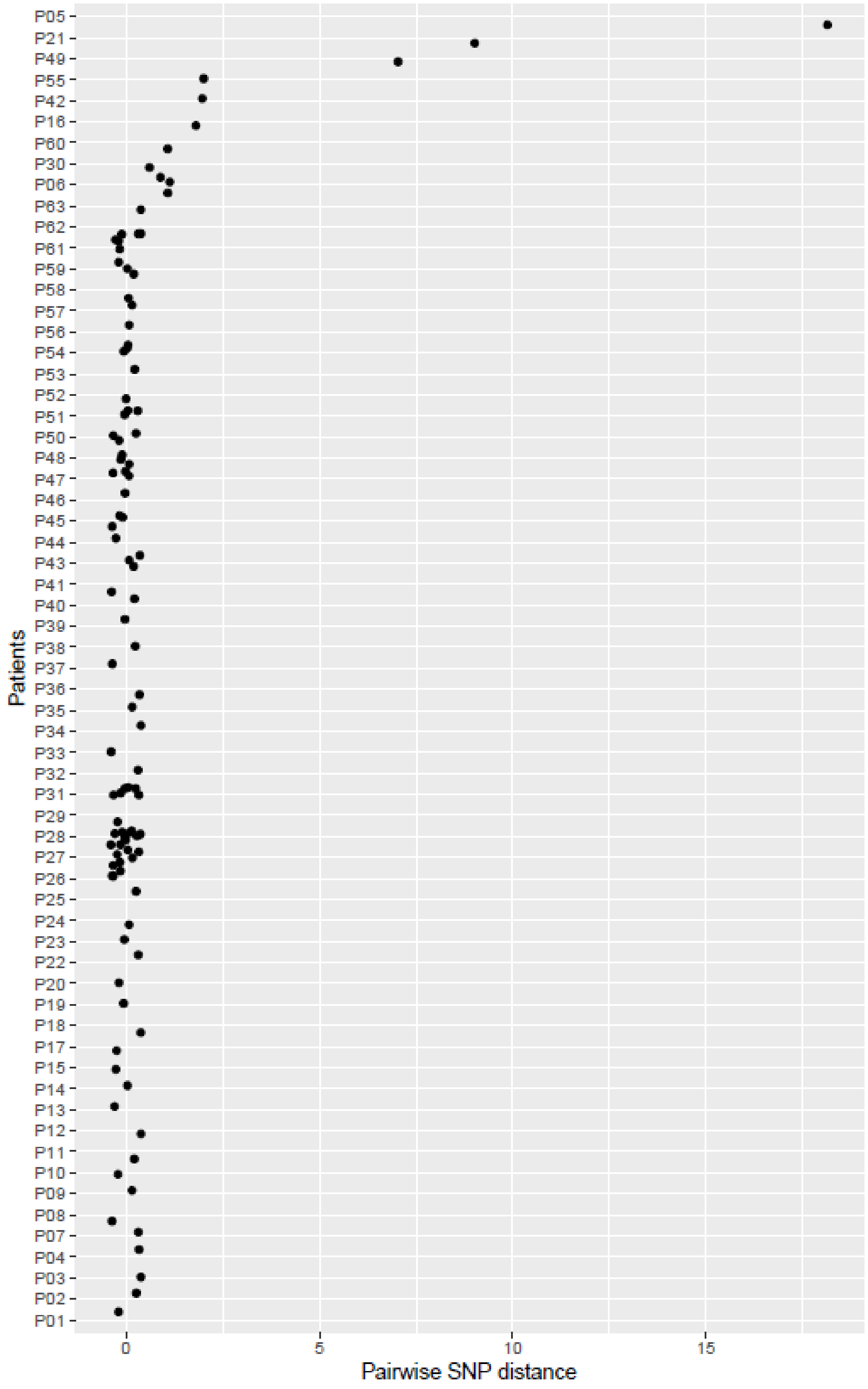

Supplementary Figure 3d. Distribution of pairwise SNP distances of samples from the same patient (within patients) and for samples from different patients (between patients)

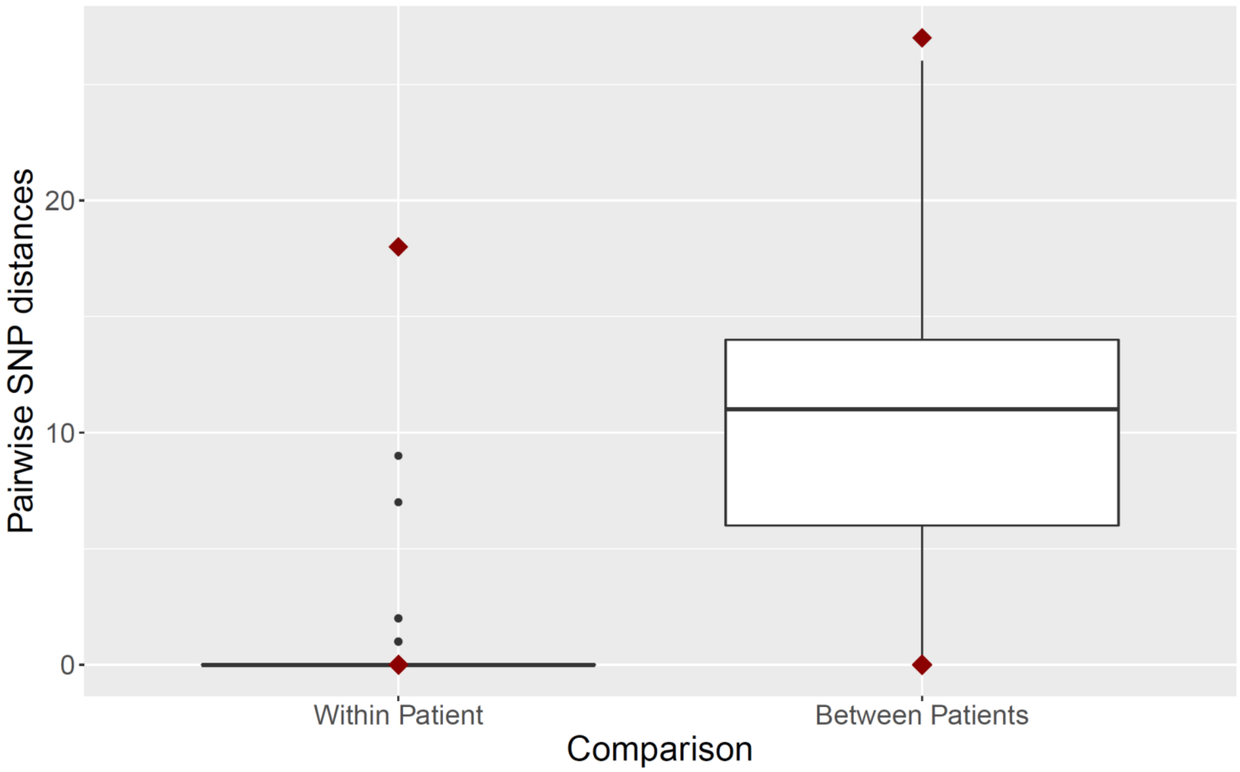

For each box in plot, middle line represents median; upper box margin represents third quartile, lower box margin represents first quartile, distance between upper and lower lines represent interquartile range (IQR), whiskers represent 1.5x IQR. Large dots represent outliers beyond 1.5x IQR. Within-patient, n=145 samples (minimum 0 SNPs, maximum 18 SNPs) from 63 patients; between patients, n=198 samples from 198 patients (minimum 0 SNPs, maximum 27 SNPs)

.

Supplementary Figure 4. Schematic figure describing inference of transmission lineages arising from imported COVID-19 cases

T, transmission lineage; S, singleton

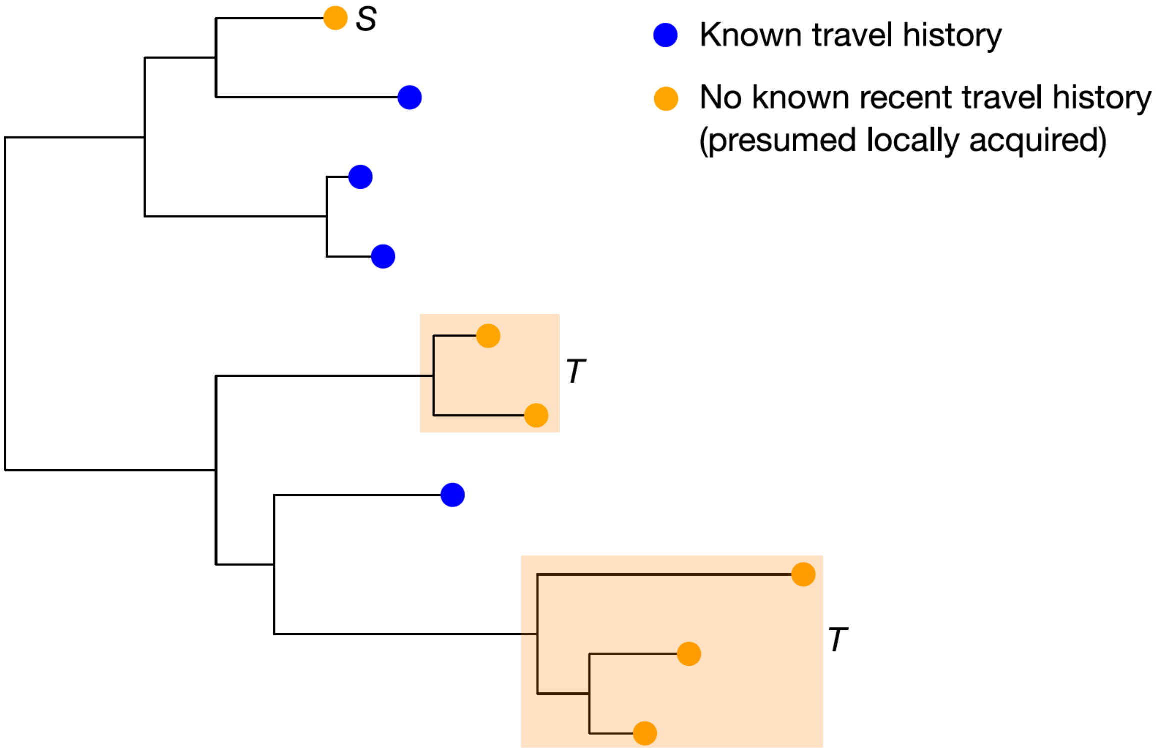

Supplementary References

1. Caly L, Druce J, Roberts J, et al. Isolation and rapid sharing of the 2019 novel coronavirus (SARS-CoV-2) from the first patient diagnosed with COVID-19 in Australia. Med J Aust 2020; 212;459-462.
2. Australian Bureau of Statistics. Standard Australian Classification of Countries (SACC), Second Edition. 2008. https://www.abs.gov.au/ausstats/abs@.nsf/mf/1269.0.
3. Li H. Minimap2: pairwise alignment for nucleotide sequences. Bioinformatics 2018;34:3094-100.
4. Li H, Handsaker B, Wysoker A, et al. The Sequence Alignment/Map format and SAMtools. Bioinformatics 2009;25:2078-9.
5. Grubaugh ND, Gangavarapu K, Quick J, et al. An amplicon-based sequencing framework for accurately measuring intrahost virus diversity using PrimalSeq and iVar. Genome Biol 2019;20:8.
6. Ondov BD, Treangen TJ, Melsted P, et al. Mash: fast genome and metagenome distance estimation using MinHash. Genome Biol 2016;17:132.
7. Howe K, Bateman A, Durbin R. QuickTree: building huge Neighbour-Joining trees of protein sequences. J Bioinformatics 2002;18:1546-7.
8. Katoh K, Misawa K, Kuma Ki, Miyata T. MAFFT: a novel method for rapid multiple sequence alignment based on fast Fourier transform. Nucleic acids research 2002;30:3059-66.
9. Minh BQ, Schmidt HA, Chernomor O, et al. IQ-TREE 2: New models and efficient methods for phylogenetic inference in the genomic era. J Molecular Biology Evolution 2020;37:1530-4.
10. FigTree: tree figure drawing tool. 2016. http://tree.bio.ed.ac.uk/
11. Ragonnet-Cronin M, Hodcroft E, Hué S, et al. Automated analysis of phylogenetic clusters. BMC Bioinformatics 2013;14:317.
12. Rambaut A, Holmes EC, Hill V, et al. A dynamic nomenclature proposal for SARS-CoV-2 to assist genomic epidemiology. Preprint at bioRxiv https://www.biorxiv.org/content/10.1101/2020.04.17.046086v1, 2020.
13. Bouckaert R, Vaughan TG, Barido-Sottani J, et al. BEAST 2.5: An advanced software platform for Bayesian evolutionary analysis. J PLoS Computational Biology 2019;15:e1006650.
14. Duchene S, Featherstone L, Haritopoulou-Sinanidou M, Rambaut A, Lemey P, Baele G. Temporal signal and the phylodynamic threshold of SARS-CoV-2. Preprint at bioRxiv https://www.biorxiv.org/content/10.1101/2020.05.04.077735v1, 2020.
15. Ho SY, Duchêne S, Duchêne D. Simulating and detecting autocorrelation of molecular evolutionary rates among lineages. Molecular ecology resources 2015;15:688-96.

16. Pybus OG, Rambaut A, Du Plessis L, et al. Preliminary analysis of SARS-CoV-2 importation and establishment of UK transmission lineages. Preprint at https://virological.org/t/preliminary-analysis-of-sars-cov-2-importation-establishment-of-uk-transmission-lineages/507, 2020.
17. Kalkauskas A, Perron U, Sun Y, et al. Sampling bias and model choice in continuous phylogeography: getting lost on a random walk. Preprint at bioRxiv 2020:2020.02.18.954057 [https://www.biorxiv.org/content/10.1101/2020.02.18.954057v1.full].
18. De Maio N, Walker C, Borges R, Weilguny L, Slodkowicz G, Goldman N. Issues with SARS-CoV-2 sequencing data. Preprint at https://virological.org/t/issues-with-sars-cov-2-sequencing-data/473, 2020.
19. Rambaut A, Lam TT, Max Carvalho L, Pybus OG. Exploring the temporal structure of heterochronous sequences using TempEst (formerly Path-O-Gen). J Virus evolution 2016;2:vew007.
20. Andersen KG, Rambaut A, Lipkin WI, Holmes EC, Garry RF. The proximal origin of SARS-CoV-2. Nature Medicine 2020;26:450-2.
21. Stadler T, Kühnert D, Bonhoeffer S, Drummond AJ. Birth–death skyline plot reveals temporal changes of epidemic spread in HIV and hepatitis C virus (HCV). J Proc Nat Acad Sci 2013;110:228-33.
22. Department of Health and Human Services Victoria. Theoretical modelling to inform Victoria's response to coronavirus (COVID-19). 2020 [https://www.dhhs.vic.gov.au/theoretical-modelling-inform-victorias-response-coronavirus-covid-19].
25. ARTIC Network. ARTIC-nCoV2019 primer schemes (Github) [https://github.com/artic-network/artic-ncov2019/tree/master/primer_schemes/nCoV-2019/V3], 2020.
