# Supplementary material for: Tracking the COVID-19 pandemic in Australia using genomics
Source: Nat Commun. 2020 Sep 1;11:4376. doi: 10.1038/s41467-020-18314-x (PMC7462846; doi:10.1038/s41467-020-18314-x)
Supplement: Supplementary file 3 — Descriptions of Additional Supplementary Files [file 41467_2020_18314_MOESM3_ESM.pdf]

Descriptions of Additional Supplementary Files

Supplementary Data 1

Description: Included sequences, sequencing information, lineages and accession numbers.

Supplementary Data 2

Description: Primer sequences for ARTIC protocol tiled amplicon PCR.

Supplementary Data 3

Description: Acknowledgement of contributors to GISAID for sequences used in Figure 2.
