# Supplementary material for: Tracking the COVID-19 pandemic in Australia using genomics
Source: Nat Commun. 2020 Sep 1;11:4376. doi: 10.1038/s41467-020-18314-x (PMC7462846; doi:10.1038/s41467-020-18314-x)
Supplement: Supplementary file 7 — Reporting Summary [file 41467_2020_18314_MOESM7_ESM.pdf]

nature research

Corresponding author(s): Benjamin Howden

Last updated by author(s): Aug 10, 2020

Reporting Summary

Nature Research wishes to improve the reproducibility of the work that we publish. This form provides structure for consistency and transparency in reporting. For further information on Nature Research policies, see our Editorial Policies and the Editorial Policy Checklist.

Statistics

For all statistical analyses, confirm that the following items are present in the figure legend, table legend, main text, or Methods section.

n/a	Confirmed	
☐	☒	The exact sample size (*n*) for each experimental group/condition, given as a discrete number and unit of measurement
☐	☒	A statement on whether measurements were taken from distinct samples or whether the same sample was measured repeatedly
☐	☒	The statistical test(s) used AND whether they are one- or two-sided<br>*Only common tests should be described solely by name; describe more complex techniques in the Methods section.*
☐	☒	A description of all covariates tested
☐	☒	A description of any assumptions or corrections, such as tests of normality and adjustment for multiple comparisons
☐	☒	A full description of the statistical parameters including central tendency (e.g. means) or other basic estimates (e.g. regression coefficient) AND variation (e.g. standard deviation) or associated estimates of uncertainty (e.g. confidence intervals)
☐	☒	For null hypothesis testing, the test statistic (e.g. F, t, r) with confidence intervals, effect sizes, degrees of freedom and P value noted<br>*Give P values as exact values whenever suitable.*
☐	☒	For Bayesian analysis, information on the choice of priors and Markov chain Monte Carlo settings
☐	☒	For hierarchical and complex designs, identification of the appropriate level for tests and full reporting of outcomes
☐	☒	Estimates of effect sizes (e.g. Cohen's *d*, Pearson's *r*), indicating how they were calculated

Our web collection on statistics for biologists contains articles on many of the points above.

Software and code

Policy information about availability of computer code

Data collection	NA
Data analysis	See Supplementary Data for settings used for all tools<br>Minimap2 v2.17<br>SAMtools v1.10<br>iVar v1.2.1<br>MASH v2.2.2<br>Quicktree v2.5<br>MAFFT v7.453<br>Arbow v0.4.0 (https://github.com/MDU-PHL/arbow)<br>IQ-Tree v1.6.12<br>FigTree v1.4.4<br>ClusterPicker v1.2.3<br>Pangolin tool v1.1.11 (https://github.com/hCoV-2019/pangolin), pangolin lineages 2020-05-07 (database)<br>TempEst 1.5.3<br>BEAST v2.5<br>blast v2.10.0+<br>R for statistical computing v3.5.2

For manuscripts utilizing custom algorithms or software that are central to the research but not yet described in published literature, software must be made available to editors and reviewers. We strongly encourage code deposition in a community repository (e.g. GitHub). See the Nature Research guidelines for submitting code & software for further information.

Data

Policy information about availability of data

All manuscripts must include a data availability statement. This statement should provide the following information, where applicable:

- Accession codes, unique identifiers, or web links for publicly available datasets
- A list of figures that have associated raw data
- A description of any restrictions on data availability

Consensus sequences and Illumina sequencing reads were deposited into GenBank under BioProject PRJNA613958 (Supplementary Data); a small subset were rejected by NCBI, which we are currently investigating. Consensus genome sequences and multiple-sequence alignment for ALL samples are also available from https://github.com/MDU-PHL/COVID19-paper.

Field-specific reporting

Please select the one below that is the best fit for your research. If you are not sure, read the appropriate sections before making your selection.

☒ Life sciences ☐ Behavioural & social sciences ☐ Ecological, evolutionary & environmental sciences

For a reference copy of the document with all sections, see nature.com/documents/nr-reporting-summary-flat.pdf

Life sciences study design

All studies must disclose on these points even when the disclosure is negative.

Sample size	To present a accurate understanding of the genomics of SARS-CoV-2 in our population every available samples underwent genome sequencing and was included in the study.
Data exclusions	To ensure high quality analysis, we applied quality control criteria for the sequence data. We applied quality control checks on consensus sequences, requiring ≥80% genome recovered, ≤25 single nucleotide polymorphisms (SNPs) from the reference genome, and ≤300 ambiguous or missing bases for sequences to 'pass' QC. Duplicate sequences from the same patient were excluded for the multiple sequence alignment, but used in the intra-patient variability analysis.
Replication	Ten samples were sequenced in duplicate in two different sequencing runs, with zero SNP differences found between the two sequences for all ten samples.
Randomization	Randomisation was not relevant to this study as all samples were subjected to the same genomic analyses in order to fully characterise the dataset.
Blinding	Scientists performing phylogenetic and phylodynamic analyses were blinded to the patient and epidemiologic data, such that genomic cluster determination was performed without knowledge of epidemiologic clustering.

Reporting for specific materials, systems and methods

We require information from authors about some types of materials, experimental systems and methods used in many studies. Here, indicate whether each material, system or method listed is relevant to your study. If you are not sure if a list item applies to your research, read the appropriate section before selecting a response.

Materials & experimental systems

n/a	Involved in the study
☒	☐ Antibodies
☒	☐ Eukaryotic cell lines
☒	☐ Palaeontology and archaeology
☒	☐ Animals and other organisms
☒	☐ Human research participants
☒	☐ Clinical data
☒	☐ Dual use research of concern

Methods

n/a	Involved in the study
☒	☐ ChIP-seq
☒	☐ Flow cytometry
☒	☐ MRI-based neuroimaging
